# Preparation of kenaf stem hemicellulosic hydrolysate and its fermentability in microbial production of xylitol by ***Escherichia coli*BL21**

**DOI:** 10.1038/s41598-019-40807-z

**Published:** 2019-03-11

**Authors:** Siti Syazwani Mohd Shah, Abdullah Amru Indera Luthfi, Kheng Oon Low, Shuhaida Harun, Shareena Fairuz Abdul Manaf, Rosli Md. Illias, Jamaliah Md. Jahim

**Affiliations:** 1 Chemical Engineering Programme, Faculty of Engineering and Built Environment, Universiti Kebangsaan Malaysia, 43600 Bangi, Selangor Darul Ehsan Malaysia; 2 Research Centre of Sustainable Process Technology (CESPRO), Faculty of Engineering and Built Environment, Universiti Kebangsaan Malaysia, 43600 Bangi, Selangor Darul Ehsan Malaysia; 3 Faculty of Chemical Engineering, Universiti Teknologi MARA, 40450 Shah Alam, Selangor Darul Ehsan Malaysia; 4 Malaysia Genome Institute (MGI), National Insitutes of Biotechnology Malaysia, Jalan Bangi Lama, Kajang, Malaysia

## Abstract

Kenaf (*Hibiscus cannabinus L*.), a potential fibre crop with a desirably high growth rate, could serve as a sustainable feedstock in the production of xylitol. In this work, the extraction of soluble products of kenaf through dilute nitric-acid hydrolysis was elucidated with respect to three parameters, namely temperature, residence time, and acid concentration. The study will assist in evaluating the performance in terms of xylose recovery. The result point out that the maximum xylose yield of 30.7 g per 100 g of dry kenaf was attained from 2% (v/v) HNO_3_ at 130 °C for 60 min. The detoxified hydrolysate was incorporated as the primary carbon source for subsequent fermentation by recombinant *Escherichia coli* and the performance of strain on five different semi-synthetic media on xylitol production were evaluated herein. Among these media, batch cultivation in a basal salt medium (BSM) afforded the highest xylitol yield of 0.35 g/g based on xylose consumption, which corresponded to 92.8% substrate utilization after 38 h. Subsequently, fermentation by *E*. *coli* in the xylose-based kenaf hydrolysate supplemented with BSM resulting in 6.8 g/L xylitol which corresponding to xylitol yield of 0.38 g/g. These findings suggested that the use of kenaf as the fermentation feedstock could be advantageous for the development of sustainable xylitol production.

## Introduction

Xylitol is a non-fermentable pentahydroxy sugar-alcohol found in some fruits and vegetables including mushrooms, lettuces, berries and corns^1,2^. The U.S. Food and Drug Administration (FDA) has considered xylitol as a healthy sugar substitute, given its comparable sweetness as that of regular sucrose, coupled with a 40% lower calorific value^3^. The rising public-health concerns with sugar (i.e. sucrose) have spurred the global demand for xylitol owing to its anticarcinogenicity and prebiotic nature^4^. The global production of xylitol has been reported to reach 160,000 tonnes with an estimated selling price of US$ 4–5 per kg^5^. The market value of xylitol was worth approximately US$ 670 million in 2013, with the Asia-Pacific region being the important player^6^. Thus, xylitol has been classified as a potential value-added chemicals from bio-renewable resources, according to the U.S. Department of Energy^2^.

The chemical production of xylitol involves catalytic hydrogenation of a highly purified D-xylose in the existence of Raney nickel catalysts^7^. The extreme pressures and temperatures entailed by the chemical production translate, in practice, into considerable consumption of energy and cost^8^. On the contrary, the biochemical approach through microbial cultivation constitutes a viable alternative to the chemical counterpart in the production of xylitol, owing to its lesser complexity, inherent sustainability and environmentally-friendly nature^7^.

Several fermenting bacteria capable of producing xylitol have been evaluated, including *Corynebacterium glutamicum*, *Serratia marcescens*, *Enterobacter liquefaciens*, *Cellulomonas cellulans* and *Bacillus coagulans* which incorporate xylose or xylulose as carbon substrates^9^. Previously, *Corynebacterium* sp. and *E*. *liquefaciens* were reported to obtain the maximum xylitol yield of around 0.33 g/g by incorporating 20 g/L xylose^3,10^. However, prior research involving the use of lignocellulosic material in the economical production of xylitol by bacteria is still insufficient. Only a handful of research work has been dedicated to direct utilization of crop biomass for xylitol production to replace the use of the costlier commercial sugar as the main carbon source. The abundance of genomic information associated with *Escherichia coli* (*E*. *coli*) has thus allowed the engineering of biosynthetic pathways for valuable xylitol production^11^. Given the natural occurrence of NAD(P)H-dependent xylose reductase (*xr*) gene in most wild-type yeasts and filamentous fungi such as *Candida* sp. and *Neurospora* sp., these microorganisms could directly convert xylose into xylitol efficiently^12,13^. Genetic modification of the *E*. *coli* strain through heterologous expression of the *xr* gene from various yeast sources has been found to enable xylose uptake for subsequent xylitol production^9^.

The utilization of low-cost feedstocks such as soybean stalks, sugarcane bagasse, corn cobs and wheat straw for xylitol production has been reported in the literature^2,14^. For instance, Paidimuddala and Gummadi^15^ asserted that the use of hydrolysates from biomass could afford the maximum xylitol yields of 0.30–0.32 g/g, under aerobic conditions. These agricultural residues consist of three major components: cellulose, lignin, and hemicellulose. It is important to highlight that in the past few decades, kenaf stem has been utilized for relatively lower value added productions of pulp and papers, furniture, livestock feed and bio-composite, but unfortunately, none of it has reached any of the biochemical production stage^8,16,17^. Given the manifold applications, it has been estimated that 0.5 million tonnes of kenaf was produced globally per annum^18^. Kenaf has been reported to absorb relatively higher nitrogen and phosphorus in the soil, with the average absorption rates of around 0.81 g/m^2^ per day and 0.11 g/m^2^ per day, respectively. Besides nutrient absorption, kenaf captures greenhouse gas at a significantly high rate. This is mainly because kenaf has much higher photosynthesis rate compared to some other conventional trees and could contribute to the pursuit of global sustainable development^19^. This third world fiber crop (i.e. after wood and bamboo) is endowed with inherent pest-resistant properties and a high growth rate of 180–220 kg per hectare, making it a favourable choice for cultivation as a major industrial crop in the future^20,21^. Cellulose fiber from kenaf stem has been used in bio-composite as a reinforcement material which offers a biodegradable and natural alternative to petroleum-based polymers. With the aim to produce high quality bio-composites, researcher has been focusing on removing lignin and hemicellulose because both of these components have hydrophilic properties and can result in unwanted moisture absorption when applying bio-composites in plastic materials^22^. In the world scenario, cellulose has been actively used in bio-composite manufacturing, leaving behind the hemicellulose part unused.

Fractionation of hemicellulose was required for the recovery of pentose sugars and the subsequent fermentative production of xylitol. Recently, numerous fractionation technologies for hemicellulose have been developed, such as treatment with dilute sulfuric acid^23^, ethanolic hot compressed water^24^, and potassium hydroxide-based steam explosion^25^. Despite the elucidation on the laboratory scale, such methods are associated with limitations on a large scale such as cost concerns and severity of operating conditions. Dilute nitric acid has been regarded as an effective solvent for pretreatment of hemicellulosic biomass compared to hydrochloric acid and sulfuric acid. Upon neutralization, nitric acid will be converted to nitrate, which can be incorporated in fermentation as the nitrogen source. Other advantages include long-time compatibility with stainless steel and utterly environmental friendly^26,27^. Moreover, the use of nitric acid has been proven to be superior in terms of xylose recovery^28^, as is likewise reflected in the form of high saccharification efficiency^4^. In dilute-acid hydrolysis, the mechanistic explanation underlying the selectivity in depolymerization of hemicellulose (mainly xylan) is that the weaker glycosidic bonds in the hemicellulose fraction can be targetted whereas stronger bonds in the cellulose (glucan) and lignin fractions are left unaffected^29^. In addition, it has been found that the attainment of a high-yield conversion of xylan necessitates the pretreatment under only mild conditions to prevent substantial depolymerization of cellulose^30^.

Against this background, this work aimed to optimize the recovery of xylose during the nitric-acid hydrolysis of kenaf stems by varying the key parameters such as hydrolysis temperature, residence time and the concentration of nitric acid. The maximum recoverable xylose in the hydrolysate obtained from the optimization study was further detoxified and adopted as the main carbon source for subsequent fermentation. The relative effects of different types of media on the xylitol production were also investigated in the batch cultivation of recombinant *E*. *coli* under aerobic conditions. Xylitol can be produced via two pathways, which can either directly be converted from xylose by NAD(P)H-dependent *xr* gene or reduced from xylulose by NADH-dependent xylitol dehydrogenase (*xd*) which was preceded by the isomerization of xylose by xylose isomerase (xylA). Furthermore, xylulose could also be phosphorylated to xylulose-5-phosphate by xylulokinase (xylB)^31^. Herein, we proposed that the deletion of the chromosomal xylA and xylB genes in combination with the co-expression of *xr* gene in *E*. *coli* BL21(DE3) could possibly enhance xylitol accumulation by minimizing the formation of undesired byproducts^1^. Collectively, this is the first report focusing on the biotechnological production of xylitol from kenaf hemicellulosic hydrolysate through *E*. *coli* BL21.

## Results

### The effect of temperature, residence time and nitric acid concentration on fermentable sugar production

Characterization conducted for raw kenaf showed that the contents of glucan, xylan and lignin were 40.8%, 34.2%, and 16.2%, respectively. The composition of the raw kenaf obtained in this study was compared with other previous studies as shown in Table 1. The variations in the chemical composition of the raw kenaf stem were due to the genetic variation, soil type, growth environment, plant age, and harvesting technique^32^. Ang *et al*.^33^ reported that kenaf plant should be harvested at the age of less than 5 months after planting in order to obtain high hemicellulose content in tandem with low lignin content. The purity of xylose in the hydrolysate attainable after nitric acid-based hydrolysis is 87.6%, while the highest dissolution rate of hemicellulose reaches about 89.6%. Apart from hemicellulose, glucan was also removed in small quantity with only 5.6% removal. Accordingly, the final compositions of the solid fraction of dry kenaf stem (% g/g) after hydrolysis were 38.5%, 2.9% and 14.8%, respectively. The finding signifies that most of the glucan molecule in kenaf stem remains intact, and that increases the purity of pentoses in the liquid stream.

**Table 1:** Chemical compositions of raw kenaf stem compared to other studies.

Particle size (µm)	Cellulose (Glucan)	Hemicellulose (Xylan)	Total lignin	Ash	Total extractives	Method	Reference
250–420	40.8 ± 1.6	34.2 ± 1.2	16.2 ± 1.9	0.29 ± 0.1	11.1 ± 0.5	NREL	This study
250–420	54.9 ± 0.2	28.7 ± 1.0	16.0 ± 0.2	4.4 ± 0.1	12.1 ± 0.1	TAPPI	^33^
0.2–0.5	43.0 ± 4.2	16.0 ± 1.2	17.0 ± 2.1	0.3 ± 0.1	21.0 ± 1.0	NREL LAP	^70^
N/A	46.1	29.7	22.1	1.6	3.9	TAPPI	^71^
250–420	37.3	51.8	14.4	4.7	10.0	NREL	^21^

All data are based on the average of three parallel replicates ± standard deviation.

NREL, TAPPI and LAP denote “National Renewable Energy Laboratory”, “Technical Association of the Pulp Industry” and “Laboratory Analytical Procedures”.

N/A: Not Available.

**Figure 1:**
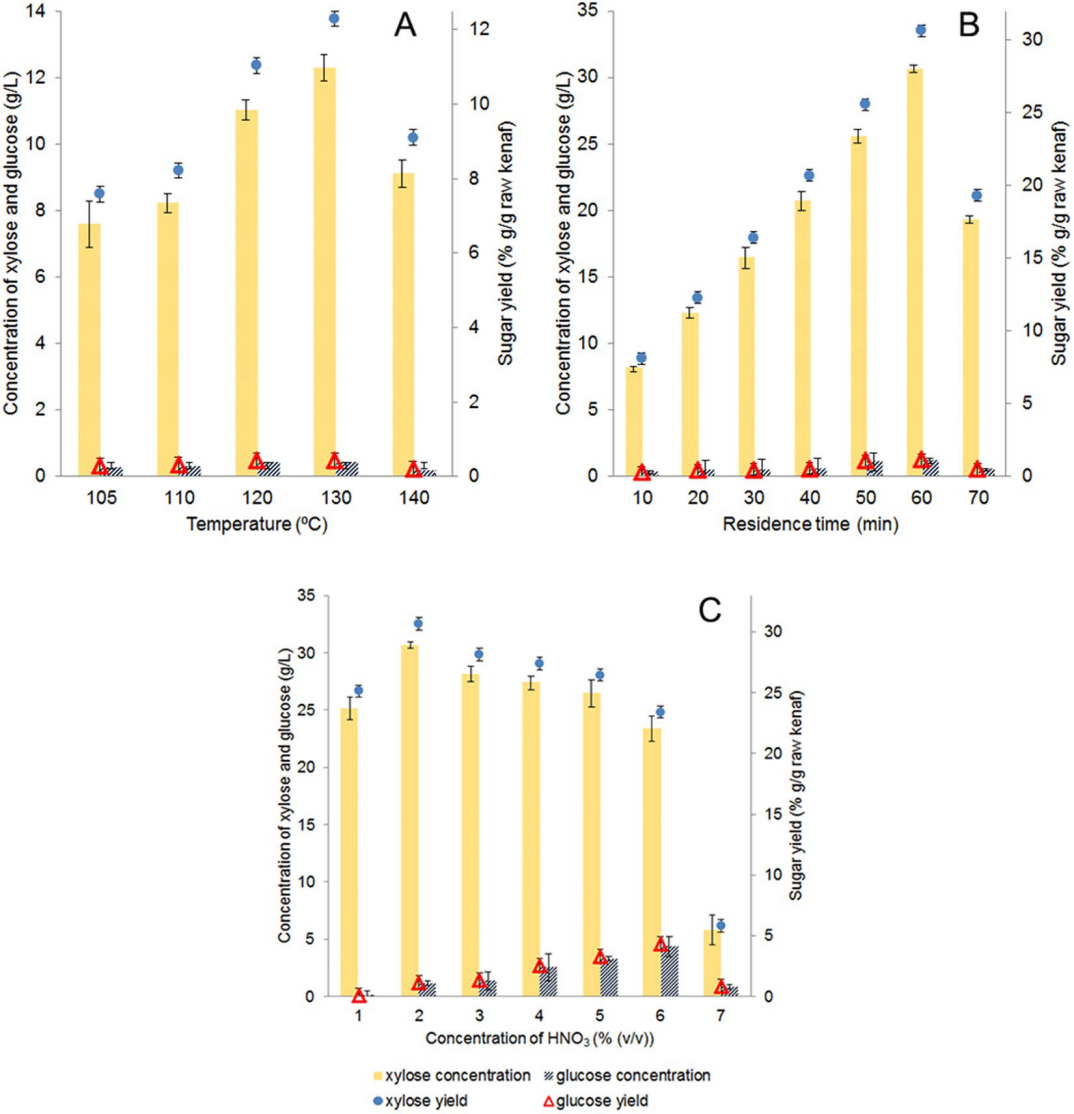
The effects of (**A**) temperature (105–140 °C), (**B**) residence time (10–70 min), and (**C**) nitric acid concentration (1–7% (v/v)) on acid hydrolysis of kenaf; xylose concentration (orange bar); glucose concentration (blue bar); xylose yield (filled circle); glucose yield (open triangle). Error bars indicate the standard deviations of three independent experiments.

The interaction between temperature, residence time and acid concentration on xylose accumulation was investigated using one-factor-at-a-time (OFAT) approach as depicted in Fig. 1. The production of xylose increased considerably with temperature until 130 °C, after which the xylose production levelled off (Fig. 1A). The highest concentration of xylose liberated from the pretreated kenaf stem in 2% (v/v) HNO_3_ for 20 min was 12.3 g/L obtained at 130 °C, which was 62% higher than that obtained at 105 °C. It was therefore recommended that the optimum temperature during nitric-acid hydrolysis should be 130 °C.

The effect of residence time on hydrolysis was further studied by maintaining the temperature at 130 °C. As could be seen in Fig. 1B, the long residence time was demonstrated to elevate the production of xylose, as it could extend more efficient solubilization of the hemicellulose fraction of kenaf. The hydrolysis lasting for 60 min yielded the best xylose concentration of up to 30.7 g/L; conversely, the hydrolysis lasting beyond this period are no longer appear to favor the production of fermentable sugars.

Apart from the residence time and temperature, the nitric acid concentration is among the driving force affecting the conversion of xylan to xylose^30^. When the acid concentrations were used at 3*–*7% (v/v), the concentration of xylose declined (Fig. 1C). The highest xylose release of 30.7 g/L occurred at 2% (v/v) HNO_3_. However, it is noteworthy that the concentration of glucose increased with nitric acid concentration. Therefore, the optimum temperature, residence time and nitric acid concentration were found to be 130 °C, 60 min and 2% (v/v) HNO_3_, respectively. The efficiency (*E*) of the acid-based hydrolysis was determined by calculating the ratio of total sugar concentration to total concentration of all inhibitors in the hydrolysates, and was expressed in unit g/g^4^. The average *E* for nitric acid hydrolysis obtained in previous studies was around 9.7 g/g^4,34,35^. Rodriguez-Chong *et al*. reported a significant *E* of 7.4 g/g for nitric acid hydrolysis under optimal conditions, compared to 5.2 g/g and 5.3 g/g for sulfuric and hydrochloric acids, respectively^4^. The extent of *E* of the process depends on the concentration of the inhibitory compounds. The high *E* value obtained in this study (10.2 g/g) was attributed to high concentration of sugars and low inhibitor formation. It is clear that the application of dilute-acid treatment at elevated temperatures for a judicious time led to the liberation of greater quantities of sugars from the lignocellulose material, which was in agreement with the previous studies^36,37^.

### Removal of fermentation inhibitors by detoxification

**Figure 2:**
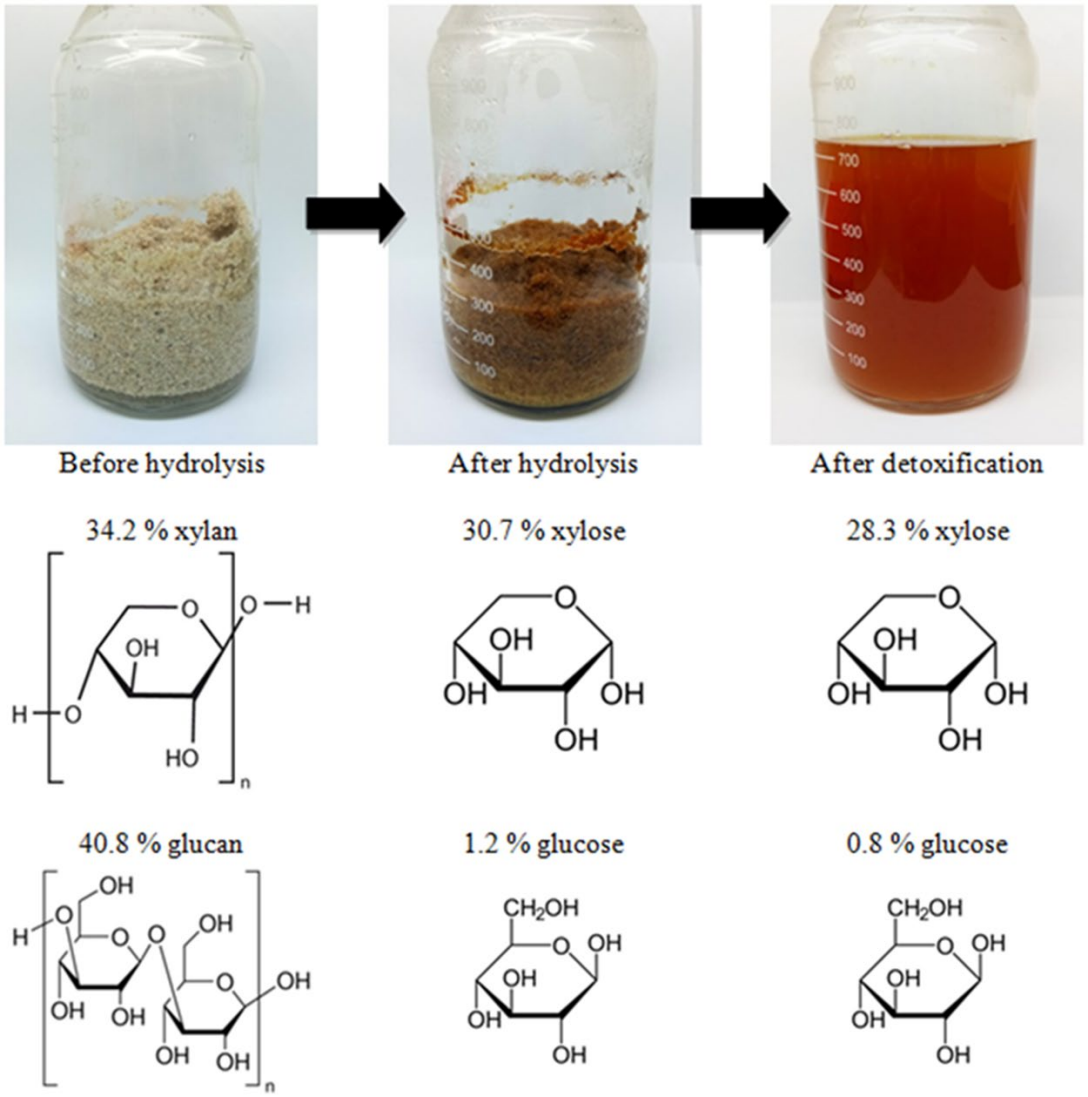
Physical appearances of kenaf before and after hydrolysis, and the hydrolysate obtained after detoxification.

All the acid-based hydrolyses inevitably gave rise to various growth-inhibitory compounds classified as phenolics, aliphatic acids, and furan derivatives^38^. Under the optimum condition, the formations of furfural (0.6 g/L) and HMF (0.03 g/L) tend to jeopardize the overall fermentative performance. Thus, detoxification was necessary before the hydrolysate could be utilized in the fermentation. Among other methods, detoxification by activated carbon is a cost-effective approach to achieve the maximum removal of furfural and phenolics^39^. Table 2 outlines the results obtained by comparing both the un-detoxified hydrolysate to the activated carbon-detoxified hydrolysate; their physical appearances are depicted in Fig. 2. This method successfully removed 66.7%, 83.3% and 4.0% of HMF, furfural, and acetic acid, respectively. Providentially, the presence of acetic acid (≥2.6 g/L) led to adverse effect during fermentation^30^.

**Table 2:** Comparison between undetoxified and activated carbon-detoxified hydrolysate.

Component	Undetoxified hydrolysate (g/L)	Detoxified hydrolysate (g/L)	% Reduction
Glucose	1.2 ± 0.2	0.8 ± 0.7	33.3
Xylose	30.7 ± 1.0	28.3 ± 1.8	7.8
Acetic acid	2.5 ± 0.3	2.4 ± 0.5	4.0
HMF	0.03 ± 0.0	0.01 ± 0.0	66.7
Furfural	0.6 ± 0.1	0.1 ± 0.2	83.3

### Morphology of kenaf fibers

**Figure 3:**
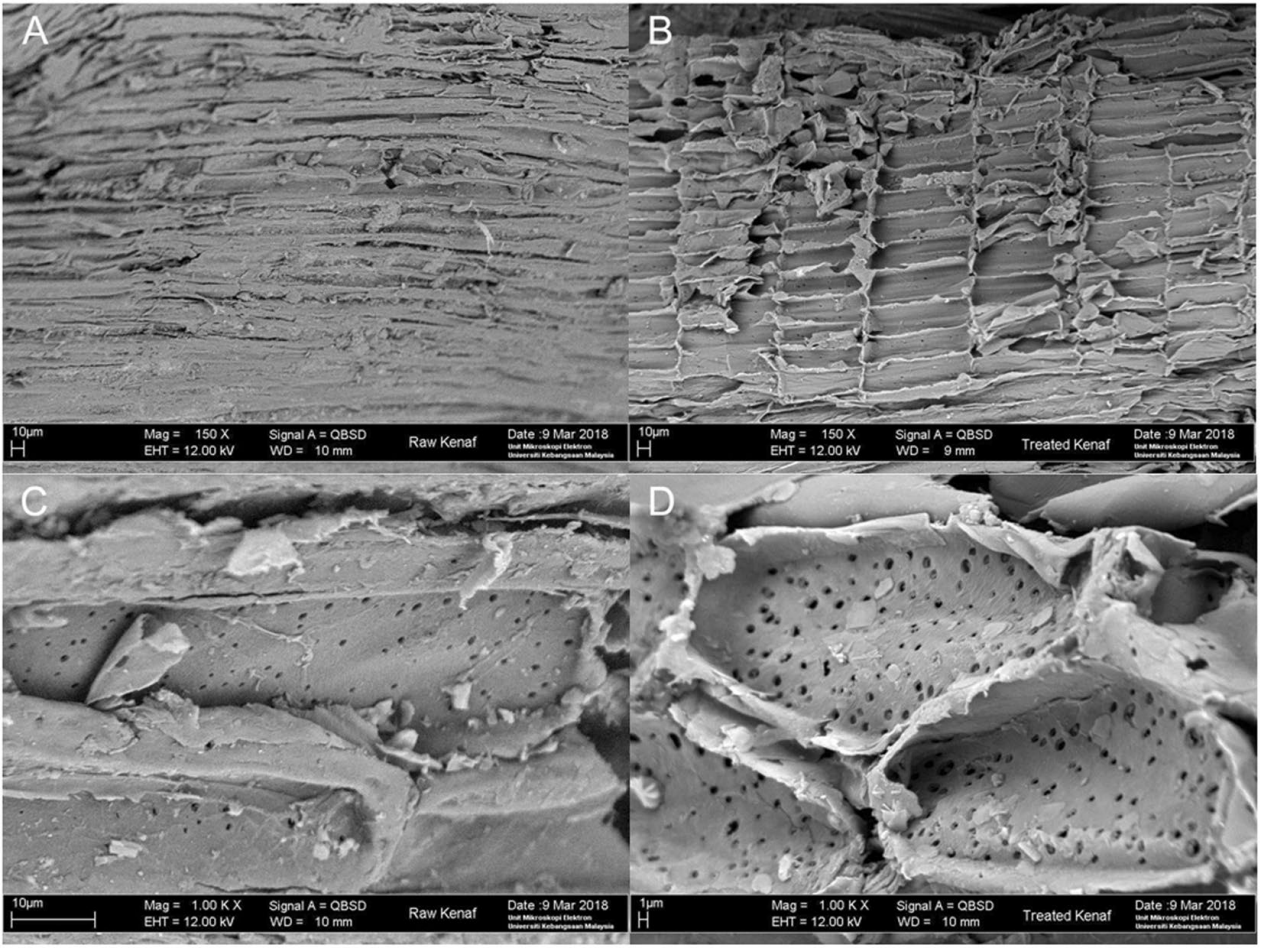
Scanning Electronic Microscope (SEM) micrographs of (**A**) surface of raw kenaf stem at a magnification of 150x, (**B**) surface of treated kenaf at a magnification of 150x, (**C**) cross-sectional view of raw kenaf stem at a magnification of 1000x and (**D**) cross-sectional view of treated kenaf with 2% (v/v) HNO_3_, at 130 °C for 60 min at a magnification of 1000x.

The morphological structures of raw and treated kenaf samples were examined through VPSEM and were presented in Fig. 3. The application of nitric acid had resulted in the roughening of the treated kenaf surfaces (Fig. 3B). Such disruption and alteration of the fibre surface were likely due to the fibre-matrix interface de-bonding and cracking when acid solutions were used to convert the natural poymer into their basic monomers^40^. Figure 3A shows that the surface structure of raw kenaf is more compact and rigid than the treated kenaf on which cracks are noted to be distributed along the alveolate surface (Fig. 3B). Furthermore, relatively small pores are noted to be present in the raw kenaf (Fig. 3C). On the other hand, when kenaf stem was subjected to nitric-acid hydrolysis, the alteration of its surface resulted in the increase of both the size and number of pores (Fig. 3D), as has been supported by previous research^41^. These phenomena are also indicative of the fact that hemicelluloses and extractives have been removed during the hydrolysis, while leaving the insoluble lignin intact^42^. The structural changes in the cell wall occurred as a result of the disruption in the crystalline structure of the treated kenaf which removed the amorphous components of the biomass^28^.

### X-ray diffraction (XRD) analysis

**Figure 4:**
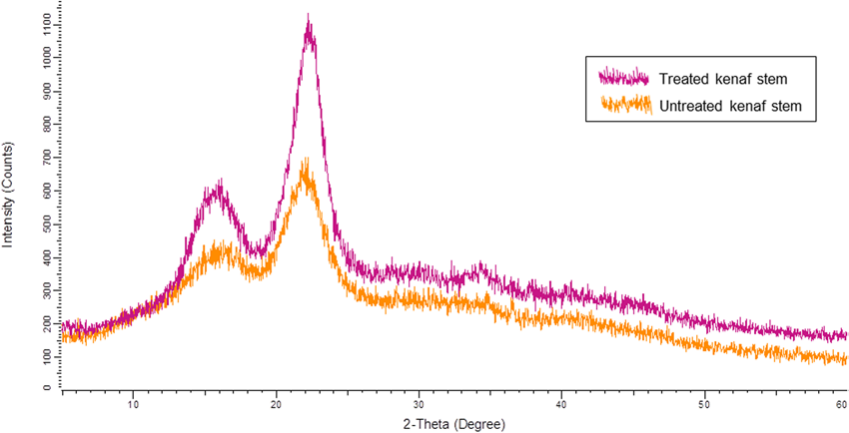
X-ray diffraction patterns for untreated and treated kenaf stem with nitric acid hydrolysis.

The crystallinity of cellulose in kenaf stem before and after hydrolysis was analysed by conducting XRD experiment as shown in Fig. 4. It could be noticed that two well-defined crystalline peaks represent amorphous at around 2θ = 16° and crystalline peaks at 2θ = 22.5°. The crystallinity index (CrI) is correlated with biomass composition, in which indicates relative amount of the crystalline cellulose in total solid^43^. The crystallinity index was found to be approximately 23.2% for the raw kenaf stem. It appears that the CrI value was increase to 31.6% after hydrolysed with dilute nitric acid. As expected, the magnitudes of these crystalline peaks increased upon hydrolysis that successfully eliminated the non-cellulosic materials in kenaf stem, which existed in the amorphous regions. The hydrolysis treatment increases the amount of cellulose exposed on the fiber surface, thereby increasing the surface roughness.

### Effect of different media on xylitol production

**Figure 5:**
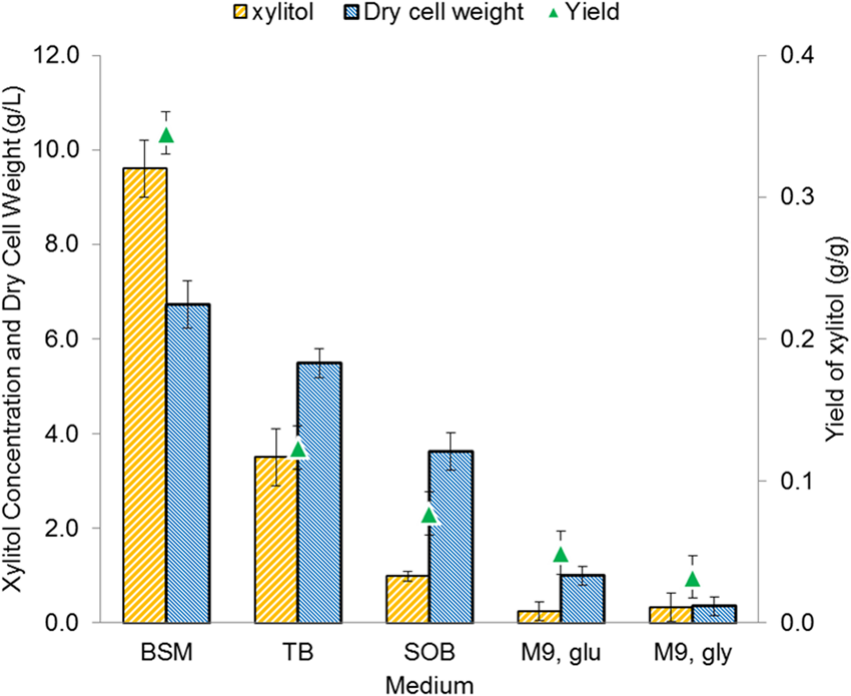
The concentrations of xylitol and biomass and the yield of xylitol for each medium screened; xylitol concentration (orange bar); cell dry weight (blue bar); xylitol yield (filled triangle). Error bars indicate the standard deviations of three independent experiments.

In this study, several fermentation media were tested for the production of xylitol using the constructed *E*. *coli* strain BL21 (DE3) ∆xylAB, p21XR. Judicious selection of the media is vital to optimise the bacterial growth and the metabolism of *E*. *coli*^44^. Figure 5 shows the performance of xylitol fermentation using BSM, TB, SOB, M9_glu_ and M9_gly_. Xylitol was only formed at 2 h following the induction by IPTG and the subsequent addition of xylose to the medium. Among all the tested media, BSM was regarded as the best medium in the production of xylitol. The maximum titer of 9.6 g/L was reached after 38 h of induction, which was about 3-times higher than that obtained in the TB medium. The final cell growth was 6.7 g/L, equivalent to OD_600_ of 19.8. After such induction, the bacterial growth remains in a stationary phase and no significant growth was observed until the end of the fermentation. Table 3 outlines the comparison of the production of xylitol using different media. In the BSM supplemented with 30 g/L of xylose, the xylitol yield of 0.35 g/g was attained. The yields of byproducts formed at the end of the fermentation using BSM were 0.05 and 0.07 g/g for ethanol and acetic acid, respectively.

**Table 3:** Comparison of xylitol production in different media. Fermentation was performed in 1-L working volume bioreactor with an initial xylose concentration of 30 g/L for 96 hours.

Medium	Fermentation duration (h)^a^	OD_600_	Dry cell weight (g/L)	Xylose consumption (%)	Xylitol Concentration (g/L)	Yield (g/g)	Productivity (g/L.h)
BSM	38	19.8	6.7	92.8	9.6	0.35	0.25
TB	24	16.2	5.5	94.4	3.5	0.12	0.15
SOB	47	10.7	3.6	42.6	1.0	0.08	0.02
M9, _glu_	52	2.9	1.0	16.7	0.2	0.06	0.01
M9, _gly_	64	1.0	0.3	34.7	0.3	0.03	0.01

^a^Time after induction with IPTG.

### Performance of kenaf hydrolysate for production of xylitol in bioreactor

**Figure 6:**
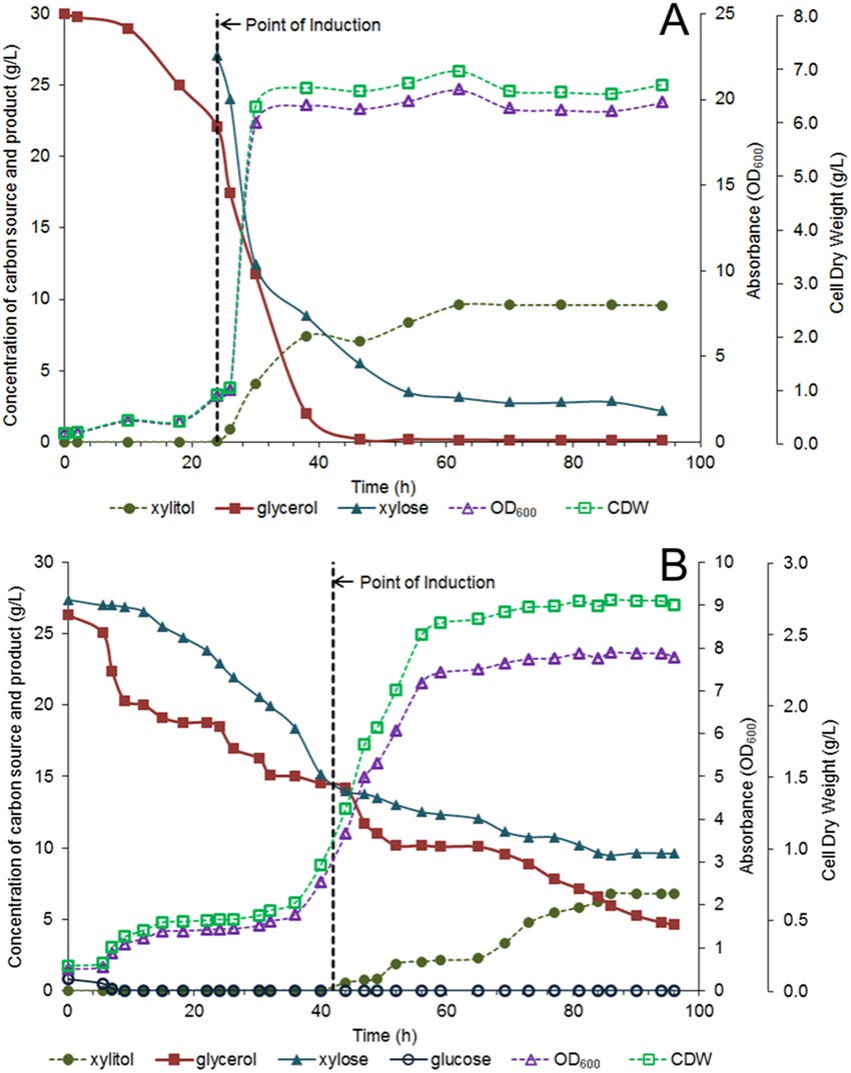
The time-course profile of batch fermentation of xylitol production by strain *E. coli* BL21 (DE3) in (**A**) BSM supplemented with pure xylose and (**B**) kenaf hemicellulosic hydrolysate; xylitol (filled circle); glycerol (filled square); xylose (filled triangle); glucose (open circle), OD_600_ (open triangle); cell dry weight (open square).

Batch fermentation was further conducted using the detoxified kenaf hydrolysate with the initial concentration of 28.2 g/L of total fermentable sugars, comprising 97.2% xylose. The established fermentation results using the hydrolysate of kenaf were compared with that using the synthethic medium, *i*.*e*., BSM suplemented with glycerol and xylose. Xylose was converted into xylitol after induction at 42 h. The time-course profiles of xylitol production during the fermentation of pure xylose and the hydrolysate are shown in Fig. 6A,B. As could be seen in Fig. 6B, the growth of the recombinant *E*. *coli* was sluggish before reaching 40 h of fermentation. After 44 h of aerobic batch cultivation, the cells grew to 2.7 g CDW/L which produced the final xylitol titer, yield and productivity of 6.8 g/L, 0.38 g/g and 0.15 g/L.h, respectively. In this study, the production of xylitol from kenaf hydrolysate appeared to be 29% lower than that obtained with synthetic media. There have been only few reports on xylitol production using recombinant *E*. *coli*. Chin and Cirino^45^ investigated the fermentation of xylitol by recombinant *E*. *coli* with glycolytic mutations, producing highest xylitol concentration of 18 g/L with initial glucose and xylose concentration of 38 g/L and 60 g/L, respectively. Other researchers also reported on the xylitol production by various microbes involving the use of lignocellulosic hydrolysate in batch fermentation. Most of the xylitol yields attainable from the oil palm frond, corncob, and sorgum stover hydrolysates were reported between 0.30 to 0.38 g/g, with superior productivity of 0.23 g/L.h obtained from sorghum stover hydrolysate^2,15,28,46^. Both studies by Paidimuddala and Gummadi^15^ and Misra *et al*.^46^ have reported comparable xylitol productivity from corn cob hemicellulosic hydrolysate, which was in the vicinity of 0.15 g/L.h. However, the maximum xylitol yield reported in this study was relatively higher than that obtained using corn-based hydrolysate, indicating that the use of kenaf as the fermentation feedstock could be advantageous in the economical production of xylitol.

## Discussion

Efficient conversion of lignocellulosic biomass to fermentable sugar has been widely reported in previous study for synthesis of renewable bio-based products^23^. In this study, acid-based hydrolysis method was used to convert kenaf to xylose-rich hydrolysate, which was then incorporated as the main carbon source in fermentative production of xylitol by *E*. *coli*. The use of dilute-nitric acid as the catalyst in the hemicellulosic hydrolysis is advantageous as it not only offers mild or moderately severe treatment but also could serve as the nitrogen source for the microorganisms during fermentation to produce xylitol^6^. The hydrolysis temperature, residence time and acid concentration are among the main factors governing the effectiveness of the nitric-acid treatment for subsequent liberation of xylose^28^. Interestingly, the highest xylan conversion (>80%) can be acquired with the use of high temperature (130 °C) and longer residence time (60 min). This was plausibly due to the dehydration of xylose to furfural in an acidic solution at elevated temperatures. In addition, longer residence time was demonstrated to elevate the production of xylose, as it could extend more efficient solubilization of the hemicellulose fraction of kenaf. In order to obtain high concentration of xylose, the hydrolysis process needs a low nitric acid concentration which is recorded at 2% (v/v). This is due to the fact that the degradation of cellulose requires more severe conditions, compared to hemicellulose, in order to completely hydrolyze its crystalline structure^30^. Hydrolysis with nitric acid concentrations ranging from 0.2 to 8.0% are considered mild, in accordance with previous studies^4,34,47^. The severity factor for nitric acid hydrolysis used in this study was calculated according to the equation described by Lee and Jeffries^48^ and was determined as 0.97, which is comparatively low. According to Hsu *et al*., yields of fermentative inhibitors such as furfural (degradation product of xylose), hydroxymethylfurfural (degradation product of glucose), and acetic acid^6,36,38^ increased as pretreatment severity increased^49^.

In addition to advance the effectiveness of the hydrolysate as a feedstock, detoxification and neutralization process were performed. The phenolics are produced from the breakdown of lignin, while the aliphatic acids are generated from the de-acetylation of hemicellulose or the breakdown of HMF^6^. The minimum microbial tolerance to phenolics is usually not more than 0.1 g/L and that to aliphatic acids is usually not more than 1.0 g/L^50^. After the dilute nitric acid hydrolysis process, the percentage of the lignin in treated kenaf stem was verified to only reduce about 8% compared to raw kenaf stem and the phenolics in the hydrolysate are too low to detect which was believed to be loss during the process. Hence, detoxification by activated carbon could be deemed as an efficient treatment for the removal of the inhibitors in kenaf hydrolysate. In light of the analysis done by other researchers, the limited consumption of xylose presumably reflected the presence of fermentation inhibitors in the medium. The increasing in xylitol yield is directly corresponded with the removal of fermentation inhibitors regardless of any technique used^28^.

**Figure 7:**
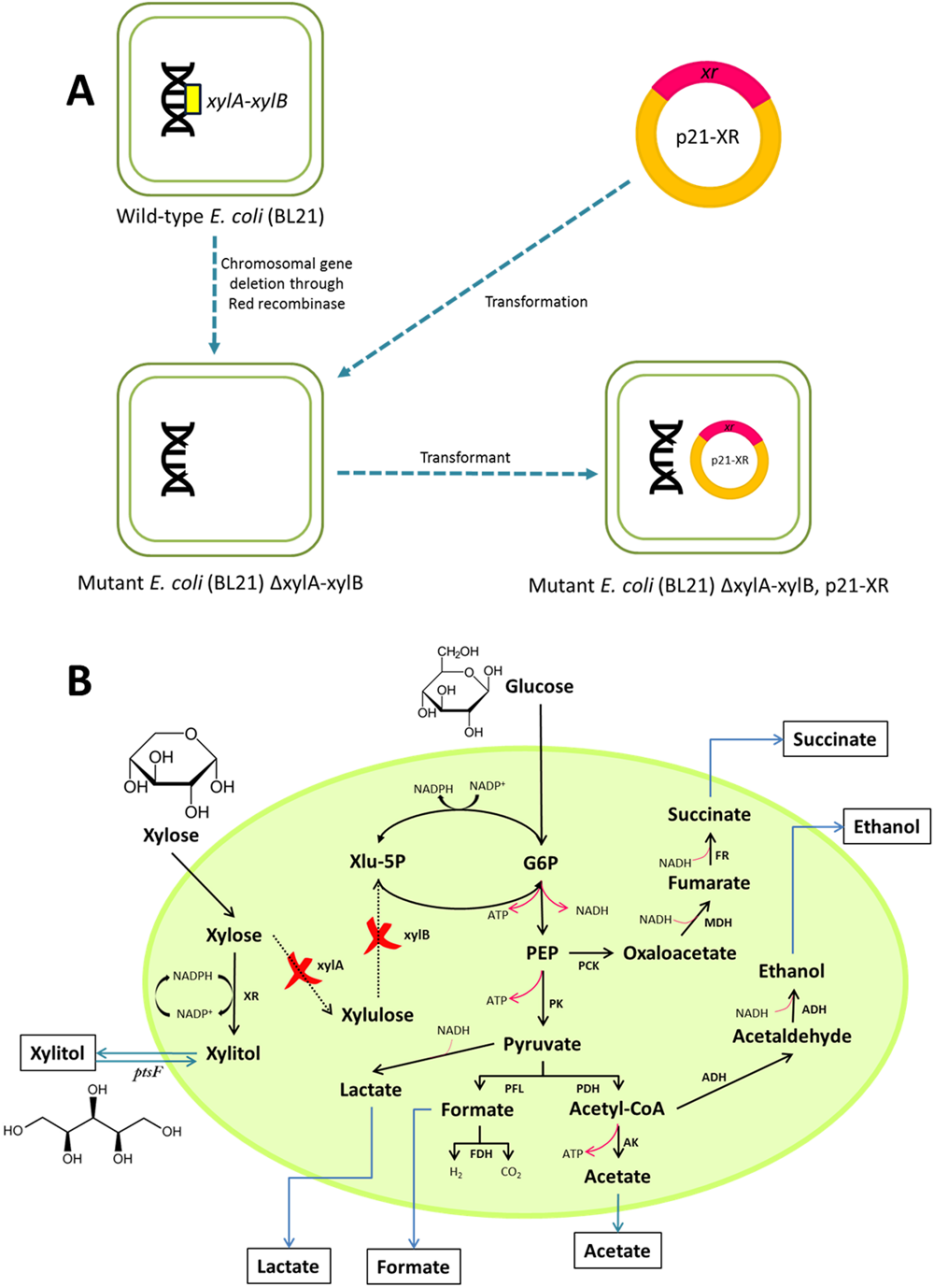
Schematic diagram of (**A**) construction of mutant *E. coli* strain BL21 (DE3) ∆xylAB, p21XR and (**B**) its plausible metabolic pathway for xylitol production from xylose-based hydrolysate. Xlu-5P, xylulose-5-phosphate; G6P, glucose 6-phosphate; PEP, phosphoenolpyruvate; PGK, phosphoglycerate kinase; PCK, phosphoenolpyruvate carboxykinase; PK, pyruvate kinase; MDH, malate dehydrogenase; FR, fumarate reductase; PDH, pyruvate dehydrogenase; FL, pyruvate-formate lyase; FDH, formate dehydrogenase; AK, acetate kinase; ADH, alcohol dehydrogenase; XR, xylose reductase^68,69^.

To date, numerous microorganisms have been major players in producing bio-based chemicals from lignocellulosic biomass including genetically modified strains^7^. Wild-type *E*. *coli* strains are unable to synthesize xylitol from xylose due to the absence of *xr* gene; instead they consume xylose through pentose phosphate pathway which eventually channels the carbon flux to tricarboxylic acid (TCA) cycle. In this study, we remove competing pathway for xylose by deleting chromosomal xylA and xylB to prevent loss of xylose to TCA cycle and transformed a recombinant plasmid-carrying *xr* gene from *Neurospora crassa*. Figure 7A overviews the recombinant *E*. *coli* strain construction. Mutant *E*. *coli* was constructed to specifically enable the uptake of xylose to form xylitol, as illustrated in Fig. 7B. This could be achieved through heterologous expression of *xr*. Previous study on *E*. *coli* has demonstrated that the uptake of xylose and the productivity for xylitol were significantly enhanced after removing the xylA and xylB genes which are responsible for the conversion of xylulose-5-phosphate from xylose^7^. Subsequently, the strain could no longer utilize xylose through pentose phosphate pathway, thereby eliminating the possible formation of by-products, such as lactate, formate, ethanol, acetate and succinate. However, the yield of xylitol obtained in this study was such lower despite the deletion of the aforementioned genes, possibly due to the intracellular accumulation of xylitol in the form of xylitol-phosphate, which might induce toxicity to the fermenting bacteria to eventually lower the production of xylitol^31^. Accordingly, several researchers have proposed to increase the yield of xylitol by removing phosphoenolpyruvate-dependent fructose phosphotransferase (*ptsF*) system which favoured the phosphorylation of xylitol to xylitol phosphate^7,31^. The resulting mutant cell was named *E*. *coli* BL21 (DE3) ΔxylAB, p21XR. Isopropyl-β-D-1-thiogalactopyranoside (IPTG) was used to induce the expression of *xr* in *E*. *coli* owing to the sulfur-carbon bond that restricted its catabolism, thereby maintaining its concentration throughout the fermentation. Accordingly, IPTG is a non-fermentable analogue of allolactose that could play a similar role by turning on the transription of the *lac operon*^51^.

Batch fermentation was performed in a bioreactor to investigate the effects of medium composition on cell growth and xylitol production in recombinant *E*. *coli*. The composition of fermentation medium can directly affect the growth and metabolism of *E*. *coli*. Enhanced metabolite production could be achieved by supplementing a proper amount of carbon and nitrogen sources in the medium to be fed to the bioreactor system. Apart from that, types of nutrient and their respective concentrations in the medium play a major role in biochemical processes, as limited supply of an essential nutrient can restrict the growth of microbial cells or product formation. Generally, the extent of metabolite production is governed by the availability of carbon and nitrogen sources in the medium^52^. Given the richness in nutrients, the media were selected specifically as they have been proven to be useful in many studies involving microbiological culture^53^, especially on *E*. *coli* cultivation^54^. The fermentation product in each medium evidently depended on the cell growth^55^. Accordingly, the concentration of xylitol decreased with the decreasing biomass content for all the media evaluated. Among the media studied, BSM shows a superior performance compared to others. This might be due to the supplementation of mineral salts and high concentration of glycerol (30 g/L) as addition to carbon sources. Both TB and SOB are complex media consisting of tryptone and yeast extract (Table 4), and the addition of glycerol to the TB medium could promote greater cell growth^55^. While in M9 medium supplemented with glycerol (M9_gly_), after prolongation of the fermentation to ~100 h, the xylitol and biomass concentrations reached about 0.3 g/L and 0.4 g/L, respectively, which were slightly higher than those in the M9 medium supplemented with glucose (M9_glu_). The overall xylose consumption in M9_gly_ was superior to that in M9_glu_, because the uptake of xylose in M9_glu_ started only after all glucose was depleted, resulting in the lower yield of xylitol. This indicated that the cells could not consume glucose and xylose simultaneously^56^. Thus, BSM was deemed successful for the optimal growth of recombinant *E*. *coli* and high productivity of xylitol, followed by TB, SOB, M9_gly_, and M9_glu_.

**Table 4:** Compositions of different types of media used for xylitol production.

Medium	Carbon source (g/L)	Nitrogen source (g/L)	Other minerals supplemented (g/L)
BSM	Glycerol (30)	NH_4_Cl (1)	KH_2_PO_4_ (3.0), Mg_2_SO_4_ (0.5), Na_2_HPO_4_ (12.8), NaCl (0.5), CuSO_4_ (6.0), NaI (0.08), MnSO_4_ (3.0), Na_2_MoO_4_ (0.2), H_3_BO_3_ (0.02), COCl_2_ (0.5), ZnCl (20), Fe_2_SO_4_ (65), H_2_SO_4_ (5), D-Biotin (0.2)
TB	Glycerol (4)	Tryptone (12), yeast extract (24)	KH_2_PO_4_ (1.16), K_2_HPO_4_ (6.27)
SOB	N/A	Tryptone (20), yeast extract (5), NH_4_Cl (5)	NaCl (0.5)
M9, glu	Glucose (5)	NH_4_Cl (1)	Na_2_HPO_4_ (12.8), KH_2_PO_4_ (3.0), NaCl (0.5), MgSO_4_ (0.493), CaCl_2_ (0.0147)
M9, gly	Glycerol (5)	NH_4_Cl (1)	Na_2_HPO_4_ (12.8), KH_2_PO_4_ (3.0), NaCl (0.5), MgSO_4_ (0.493), CaCl_2_ (0.0147)

N/A: Not Available.

Performance of kenaf hydrolysate for production of xylitol was investigated to compare with fermentation in BSM. The resulting recombinant strains exhibited an inferior growth rate and poor substrate utilization when grown on kenaf-based hydrolysate, compared to its synthetic medium. It appeared that the cells required some time to adapt in the hydrolysate. The measure of lag phase period was about two times longer for the fermentation using hydrolysate than that in the synthetic medium. This observation may be attributed to the presence of furfural, HMF and acetate in the hydrolysate at early stage of the fermentation, which exerted some inhibitory effects on the cell growth and on the xylitol production^57^. Generally, xylose fermentation by *E*. *coli* BL21 (DE3) produced xylitol as the main product. At the initial state of fermentation, glucose was the preferred carbon source and was consumed at a high rate. This confirmed the presence of the repression of carbon catabolite which prevented the uptake of both xylose and glycerol before glucose was completely depleted^58^. However, this phenomenon was advantageous, particularly for the fermentation involving the hydrolysate, as it could minimize the uptake of xylose prior to the induction with IPTG.

Nevertheless, xylitol production via a biotechnological process can be enhanced by adaptive response of the microorganism to the presence of the toxic compounds. Strain improvement strategies such as directed evolution can be employed to enhance the performance of recombinant *E*. *coli* in the fermentation^6^. The utilization of inexpensive and non-food lignocelluloses can mitigate the problem of waste disposal by converting waste into value-added products such as xylitol. Bioconversion of xylitol from lignocellulosic biomass using engineered strain of microorganism offers significant advantages for the emerging biorefinery industry. Our study elucidated the hydrolysis of xylans in kenaf with nitric acid followed by detoxification and its utilization as the feedstock in fermentation. The strain engineered was able to utilize the hydrolysate as the substrate for xylitol production. Since the formation of inhibitors in hydrolysate were minimised through detoxification, the result indicated that *E*. *coli* BL21 (DE3) were well grown in kenaf-based hydrolysate with maximum xylitol concentration and productivity of 6.8 g/L and 0.15 g/L.h, respectively.

## Methods

### Raw material preparation

Freshly-harvested kenaf stems were obtained from a local kenaf-processing company, Everise Crimson Sdn. Bhd., Bachok, Kelantan, Malaysia. The stem was washed thoroughly with deionized water, and dried at 50 °C for 48 h. Each of the dried kenaf stem, consisting of core and bast, was cut into smaller pieces before being ground in a pulverizer (Pulverisette 19, Fritsch, Germany). The ground kenaf samples were sieved mechanically to remove the adhered powders, so that only particles measuring 250–420 µm were used in this study. Subsequently, the samples were dried in an oven at 105 °C overnight before being stored in a tightly-sealed desiccator until further use^21^.

### Kenaf compositional analysis

A comprehensive analytical procedure allows the accurate characterization of lignocellulose components to be used in biochemical processes^59^. The compositional analysis of the untreated and dilute acid-treated kenaf stems was conducted according to the National Renewable Energy Laboratory (NREL, Colorado) protocol to determine the total structural carbohydrates, Klason lignin, ash content, and total extractives^60^. Initially, the kenaf samples were oven-dried to a constant moisture content lower than 10 wt% as described by Sluiter and Sluiter^61^. The Soxhlet extractor was used to extract both water-soluble components (reflux rate of 4–5 siphon cycles per hour) and ethanol-soluble ones (6–10 siphon cycles per hour) from the kenaf for 24 h. The extractive-free components of the kenaf were subjected to an autoclaved-based acid hydrolysis. Subsequently, the hydrolysate was subjected to vacuum filtration to separate the filtrate (soluble sugar monomers) from the Klason lignin^62^.

### Dilute nitric acid hydrolysis and detoxification

The effects of xylose recovery and inhibitory formation upon dilute-nitric acid hydrolysis on the kenaf stems were evaluated at the temperatures of 105–140 °C, residence times between 10–70 min and nitric acid concentrations ranging from 1–7% (v/v). The basis of selection for these ranges of parameters lies in the findings from a previous study on achieving hydrolysis under mild conditions^30^. The hydrolysis was performed in a 1-L Schott glass bottle with a liquid-to-biomass ratio of 10:1. The resultant hydrolysate was filtered through a gauze cloth to remove the un-hydrolyzed solid residue. Subsequently, coconut shell granular activated carbon (Concepts Ecotech, Malaysia) was used to detoxify the kenaf hydrolysate at 50 °C for 60 min at an agitation speed of 120 rpm^39^. The detoxified hydrolysate was further adjusted to pH 7 using 5 M NaOH for subsequent fermentative production of xylitol.

### Morphology determination

The morphologies of both raw and acid-treated kenaf under the optimized condition were examined with a scanning electron microscope (VPSEM LEO 1450, United Kingdom). Both samples were oven-dried before being mounted on aluminium stubs. The samples were then coated with palladium by plasma sputtering to avoid sample surface charging^63^.

### X-ray diffraction analysis

X-ray diffraction (XRD) was used to study the changes in structures of kenaf stem before and after hydrolysis. The crystallinities of the samples were analysed using an x-ray diffractometer (Bruker AXS D8 Advance, USA) with Ni-filter CuKα radiation (λ = 1.541 Å) at 40 kV and 40 mA. The samples were scanned at a speed of 0.25° per second with diffraction angle, 2θ = 0° to 60°^28,43^. The crystallinity index (CrI) of sample was calculated based on the diffraction intensities of crystalline and amorphous fraction by making use of the following equation:

$${\rm{Crystallinity}}\,{\rm{index}}\,({\rm{CrI}})=\frac{{I}_{002}-{I}_{am}}{{I}_{002}}\times 100$$

where I_002_ is the intensity of crystalline fraction of 002 plane at 2θ = 22.5° and I_am_ is the intensity of amorphous fraction at 2θ = 18.0°^23^.

### Construction of xylitol-producing recombinant *E*. *coli* strain

Xylose reductase (*xr*, EAA34695.1) from *Neurospora crassa* was gene synthesized and cloned into NdeI and BamHI of plasmid pET21, resulting in recombinant plasmid p21XR. Deletion of chromosomal xylA and xylB was accomplished using red recombinase, as described by Datsenko and Wanner^64^. Briefly, primers xylAB_Del_F (ATGCAAGCCTATTTTGACCAGCTCGATCGCGTTCGTTATGTGTAGGCTGGAGCTGCTTC) and xylAB_Del_R (TTACGCCATTAATGGCAGAAGTTGCTGATAGAGGCGACGATGGGAATTAGCCATGGTCC) were used to amplify FRT-flanked kanamycin resistance cassette from plasmid pKD4. The fragments acquired were transformed in *E*. *coli* competent cell carrying the Red recombinase expression vector pKD46 via electroporation, and integrated into its chromosome. Successfully disrupted colonies were then transformed with plasmid pCP20 and induced at 42 °C to eliminate the kanamycin resistance. Polymerase chain reaction (PCR) verifications were performed using primer pairs designed according to the sequences up- and downstream of disrupted regions [xylAB_DelIden_F (CATGAGATCCATAGCCCAACC) and xylAB_DelIden_R (TACCCAGTTTCATCATTCCATT)]. Hence, the resulting strain was named *E*. *coli* BL21 (DE3) ΔxylAB. This mutant strain was then transformed with plasmid p21XR, resulting *E*. *coli* BL21 (DE3) ΔxylAB, p21XR^65^ which was used for xylitol production from kenaf hydrolysate.

### Culture strain and inoculum preparation

*E*. *coli* strain BL21 (DE3) ∆xylAB, p21XR was maintained at −80 °C in 15% glycerol solution for long-term storage. The strain was routinely cultured at 37 °C on Luria-Bertani (LB) agar containing the following components per liter of distilled water: 10 g tryptone, 5 g yeast extract, 10 g sodium chloride, and 15 g agar. LB agar was used because it could permit fastidious growth for many bacterial species^66^. The inoculum was prepared in a two-stage growth process under similar conditions, unless indicated otherwise. First, a single colony of *E*. *coli* BL21 (DE3) ∆xylAB, p21XR was transferred into 10 mL of LB medium supplemented with 100 μg/mL of ampicillin, and the culture was incubated at 37 °C, 250 rpm for 6 h. Then, 10% (v/v) of the pre-grown culture was cultivated in 100 mL of LB medium for 18 h with the addition of 0.8% glycerol, which would contribute to higher biomass yields^67^.

### Effect of different media on xylitol production

Comparative fermentations were performed in a 2-L bioreactor (Infors HT, Switzerland) with five different semi-synthetic media to evaluate their effects on xylitol production: basal salt medium (BSM), terrific broth (TB), super optimum broth (SOB) and M9 minimal medium with either glucose or glycerol as the carbon source. These media were chosen specifically owing to their richness in nutrients that have been proven to be useful in many studies involving microbiological culture^53^, especially on *E*. *coli* cultivation^53,54^. Thus, the compositions of each media are outlined in Table 4.

Overnight culture inoculum of *E*. *coli* strain BL21 (DE3) ∆xylAB, p21XR was transferred into the medium at 10% of 1-L working volume under sterile condition. The oxygen flow rate was set at 1 vvm throughout the fermentation. For the purpose of increasing the cell density in the bioreactor, it was further cultivated at 250 rpm for 28 h or until the optical density (OD_600_) reached 1.0, corresponding to about 0.34 g/L cell dry weight (CDW). Subsequently, D-xylose that served as the precursor to xylitol was loaded aseptically at 30 g/L, and the expression of *xr* was induced by the addition of 0.1 mM isopropyl-β-D-1-thiogalactopyranoside (IPTG). The fermentative production of xylitol was conducted at 30 °C for 96 h and the pH was maintained at 7.0 ± 0.05 using 3 M NH_4_OH. Samples were taken at intervals of 2–4 h to monitor the cell growth, substrate consumption and metabolite production. The best media was selected by evaluating the highest cell density and xylitol production during the fermentation.

### Kenaf hydrolysate as precursor to xylitol

The precursor in the production of xylitol was substituted with the detoxified kenaf hydrolysate and run under the optimal growth condition established with the xylose-based medium from the previous section. All experiments were conducted in triplicates. The identical process conditions, other than the sources of precursor used, enabled a comparative investigation on the xylitol production.

### Analytical methods

The biomass was determined in terms of cell dry weight (in g/L) of the culture: the samples taken from the fermentation were centrifuged at 10,000 rpm for 10 min to separate cell pellet from the fermentation broth. The cell pellet was washed twice with distilled water and dried to a constant weight at 80 °C for 24 h. Bacterial growth, as a measurement of cells in suspension, was also recorded with optical density at 600 nm (OD_600_) in a spectrophotometer (GENESYS 10 UV, USA). From the resultant growth curve, one unit of OD_600_ equalled 0.34 g/L of cell dry weight.

Liquid samples of the acid-treated kenaf and the fermentation broth were filtered through 0.22 µm Whatman membrane syringe filters in vials prior to the quantifications of xylose, glucose, glycerol, xylitol, acetic acid and ethanol. The samples were subjected to high performance liquid chromatography (HPLC) equipped with Rezex ROA-Organic acid column (300 mm × 7.8 mm; Phenomenex, USA) and a guard column (50 mm × 7.8 mm) set at 60 °C. The mobile phase, i.e. 5 mM sulfuric acid, was eluted in an isocratic manner at a flow rate of 0.6 mL/min. Samples were injected at 10 µL volume, and the peak representing each component was detected by a refractive index detector (RID) set at 40 °C. Meanwhile, inhibitory compounds in the form of furfural and hydroxymethylfurfural (HMF) were analyzed using HPLC (Agilent 1100 series, California, USA) equipped with C18 column (150 mm × 4.6 mm; Phenomenex, USA). Each of the individual peaks was established with the ultraviolet-diode array detector (UV-DAD) at 220 nm.
